# Reverse Engineering of Vaccine Antigens Using High Throughput Sequencing-enhanced mRNA Display

**DOI:** 10.1016/j.ebiom.2015.06.021

**Published:** 2015-06-30

**Authors:** Nini Guo, Hongying Duan, Alla Kachko, Benjamin W. Krause, Marian E. Major, Philip R. Krause

**Affiliations:** Division of Viral Products, Office of Vaccines Research and Review, Center for Biologics Evaluation and Research, Food and Drug Administration, 10903 New Hampshire Ave, Silver Spring, MD 20993, United States

**Keywords:** mRNA display, Vaccine reverse-engineering

## Abstract

Vaccine reverse engineering is emerging as an important approach to vaccine antigen identification, recently focusing mainly on structural characterization of interactions between neutralizing monoclonal antibodies (mAbs) and antigens. Using mAbs that bind unknown antigen structures, we sought to probe the intrinsic features of antibody antigen-binding sites with a high complexity peptide library, aiming to identify conformationally optimized mimotope antigens that capture mAb-specific epitopes. Using a high throughput sequencing-enhanced messenger ribonucleic acid (mRNA) display approach, we identified high affinity binding peptides for a hepatitis C virus neutralizing mAb. Immunization with the selected peptides induced neutralizing activity similar to that of the original mAb. Antibodies elicited by the most commonly selected peptides were predominantly against specific epitopes. Thus, using mRNA display to interrogate mAbs permits high resolution identification of functional peptide antigens that direct targeted immune responses, supporting its use in vaccine reverse engineering for pathogens against which potent neutralizing mAbs are available.

**Research in Context:**

We used a large number of randomly produced small proteins (“peptides”) to identify peptides containing specific protein sequences that bind efficiently to an antibody that can prevent hepatitis C virus infection in cell culture. After the identified peptides were injected into mice, the mice produced their own antibodies with characteristics similar to the original antibody. This approach can provide previously unavailable information about antibody binding and could also be useful in developing new vaccines.

## Highlights

• mRNA-display/high throughput sequencing identified high affinity peptide binders to monoclonal antibody (mAb).
• The profile of selected peptides characterized the binding specificity of the selection mAb.
• Immune responses induced by selected peptides were neutralizing and epitope-focused, mimicking the selection mAb.

## 1 Introduction

As evidenced by the isolation and cloning of neutralizing monoclonal antibodies (mAbs) against pathogens like hepatitis C virus (HCV) and human immunodeficiency virus (HIV) for which licensed vaccines are not available, humans are capable of producing such antibodies (Sautto et al., 2013, Mascola and Haynes, 2013), but identification of these natural mAbs has not yet led to successful vaccine antigen design. Traditional approaches using whole pathogens (live, attenuated or inactivated) or subunit vaccines do not always induce the desired neutralizing antibodies. Identification of antigens that can induce humoral responses that mimic neutralizing mAbs would be very useful in developing improved vaccine strategies. Such antigens could be considered as priming vaccines (providing “original antigenic sin” that directs the immune response after subsequent exposures towards induction of desired neutralizing antibodies) or as components of multivalent vaccines (which could potentially induce immune responses against multiple neutralizing epitopes).

“Vaccine reverse engineering” (Burton, 2002) focuses on inferring the structure of improved immunogens based on interactions with neutralizing antibodies. While “epitope focused antigen design” (Correia et al., 2014) and other structural approaches (Kulp and Schief, 2013) show promise in identifying the three dimensional structure of antigens that could elicit mAb-like neutralizing responses, it also is recognized that the link between physical structure and landscapes recognized by mAbs can be analyzed using antigenic approaches (Dormitzer et al., 2008) that examine the binding specificity of mAbs. For the many neutralizing mAbs with unknown three-dimensional structures of antigen-antibody complexes, identification of antigens that can optimally present the critical epitopes to the immune system remains a challenge. We reasoned that a given mAb could be antigenically characterized by identifying a series of tightly binding antigens that, collectively and uniquely define the binding specificity of the antibody, and thus explored whether probing neutralizing antibodies with a high-complexity random peptide library could allow identification of optimized antigens that capture key information about a mAb of interest, holding the potential to generate immune responses that have properties similar to that of the mAb.

To increase the chance of successful antigen selection, we used messenger ribonucleic acid (mRNA) display, which generates random peptide libraries with complexity as high as > 10^13^ unique sequences (Takahashi et al., 2003). Each peptide is covalently bound to its encoding mRNA through a puromycin linkage, enabling in vitro selection of peptide libraries (ranging from 1 to over 100 amino acids) that bind to targets of interest (including antibodies) and the use of reverse transcriptase polymerase chain reaction (RT-PCR) and sequencing to determine the nucleic acid sequence corresponding to the peptides that bind the target. The high library complexity of mRNA display allows selection of extremely rare and high affinity binding partners, and its use in epitope mapping has been described (Baggio et al., 2002, Ja et al., 2005).

In the present study, we integrated mRNA display and high throughput sequencing (mRNA display-HTS) to identify peptide mimotopes that capture important information from HCV neutralizing mAb41 (Duan et al., 2012). This approach enabled high-resolution mapping of this mAb's binding specificity. MAb41 was previously obtained from mice immunized with a HCV genotype 1a (GT1a) wild type peptide fragment named peptide A (pA) (Duan et al., 2012), comprising amino acid residues 412 to 447 of the E2 protein. MAb41 neutralizes cell culture HCV (HCVcc) in a GT1a-specific manner (Duan et al., 2012). Using mRNA display-HTS to select for peptides that bind to mAb41, we hoped to identify peptide mimotopes that capture key information unique to this neutralizing antibody's antigen binding sites, potentially identifying peptide antigens that can present mAb-specific epitopes in an optimal context. While these peptide mimotopes likely will have limitations as stand-alone vaccine candidates, the identified antigens could potentially serve as optimized core epitope components in the context of scaffolds or other antigen presentation strategies. We evaluated the binding affinity of the selected peptides to the original mAb, and further investigated their ability to stimulate immune responses.

## 2 Materials and Methods

### 2.1 mRNA Display Selection

*Library construction*. Sequences of the deoxyribonucleic acid (DNA) library for mRNA display and the primers used for PCR amplification of the library were adopted from a previous publication (Ja and Roberts, 2004). The sense DNA oligonucleotide, 5′-GGG ACA ATT ACT ATT TAC AAT TAC AAT G (Trimer)_15or27_ CAG CTG CGT AAC TCT TGC GCT-3′, was synthesized by the Yale Keck lab. Its sequence contains a T7 promoter, a cytomegalovirus (CMV) translation enhancer, an ATG start codon followed by a 15 or 27-mer random region, and a constant 3′ region encoding the peptide QLRNSCA. “Trimer” represents the mixture of 20 trimer phosphoramidites, each representing one amino acid (Glen Research, Sterling, VA), and was used to increase library randomness and to avoid stop codons. The sense oligo was PCR amplified 6 cycles with the following primers: sense 5′-GGA TTC TAA TAC GAC TCA CTA TAG GGA CAA TTA CTA TTT ACA ATT AC -3′, and antisense 5′-AGC GCA AGA GTT ACG CAG CTG-3′ to produce the initial DNA template for in vitro transcription.

*mRNA–peptide fusion synthesis*. mRNA–peptide fusions were prepared as described previously (Takahashi and Roberts, 2009) with modifications. The PCR amplified library was transcribed in vitro using MEGAscript Kit (Life Technologies, Grand Island, NY), and purified using NucAway spin columns (Life Technologies). mRNA was then ligated to a 5'-phosphorylated puromycin linker (5′-A_21_[S9]_3_ACCP in which S9 = phosphoramidite spacer 9 (Glen Research) and P = puromycin (Glen Research)) by T4 DNA ligase (Invitrogen) in the presence of Splint (5′-TTT TTT TTT TTN AGC GCA AGA GT-3′, N = A, T, G or C) to produce puromycin-conjugated mRNA templates. The ligated library was purified by electrophoresis through a TBE-Urea Gel (Life Technologies) followed by extraction by electro-elution (D tube, Novagen, Madison, WI). The purified mRNA library was translated using the Retic Lysate IVT In Vitro Translation Kit (Life Technologies). KCl (500 mM final) and MgCl_2_ (60 mM final) were added after the translation reaction to promote fusion formation. mRNA–peptide fusions were purified by binding to Dynabeads Oligo-[dT]_25_ (Life Technologies) in 100 mM Tris (pH 7.5), 1 M NaCl, and 0.2% Triton X-100 at 4 °C. After washing, fusions were eluted at 65 °C with H_2_O. Fusions were reverse-transcribed using SuperScript First-Strand reverse transcriptase (Life Technologies).

*Selection*. After reverse-transcription, mRNA–peptide fusions were pre-cleared three times using Dynabeads Protein G (Life Technologies). Pre-cleared samples were then incubated with Dynabeads Protein G coupled with monoclonal antibodies in 1 × binding buffer (PBS plus 0.1% Triton), 0.5 mg/ml BSA (Life Technologies) and 1 μg/ml yeast tRNA (Life Technologies) for 1 h at room temperature followed by washing six times. cDNA was amplified by PCR using the sense and antisense primers listed in *Library construction*.

### 2.2 High Throughput Sequencing (HTS) and Data Analysis

PCR products from the selection step were purified using Agencourt AMPure XP beads (Beckman Coulter, Brea, CA), eluted with 10 mM Tris (pH 7.5), and subjected to 6 cycles of PCR to add Illumina adapters with the following primers: forward 5′-AAT GAT ACG GCG ACC ACC GAG ATC TAC ACT CTT TCC CTA CAC GAC GCT CTT CCG ATC TNN NNG GAT TCT AAT ACG ACT CAC TAT AG-3′ (N = A, T, C or G), and reverse 5′-CAA GCA GAA GAC GGC ATA CGA GAT CGT GAT GTG ACT GGA GTT CAG ACG TGT GCT CTT CCG ATC AGC GCA AGA GTT ACG CAG CTG-3′. PCR products were purified using Agencourt AMPure XP beads (Beckman Coulter) and were analyzed using an Agilent 2100 Bioanalyzer. 2 nanomoles of purified PCR products were denatured and subjected to MiSeq (Illumina, San Diego, CA) sequencing. Sequences were translated into peptide sequences. Sequences ending with QLRNSCA were selected and ranked by their copy numbers. A series of in-house Python programs were developed for sequence analysis.

### 2.3 Peptides

All peptides were synthesized either by the Core Facility of the Center for Biologics Evaluation and Research at the U.S. Food and Drug Administration, or by United Biosystems, Inc. (Herndon, VA). Amino acids in peptide sequences are described using single letter codes. A slash (/) denotes the alternative use of the amino acids directly before and after the slash. In some cases, a dash (–) is introduced between adjacent amino acids to simplify presentation of alignments.

### 2.4 ELISA

Biotinylated peptide enzyme-linked immunosorbent assays (ELISAs) were performed as previously described (Major et al., 1999) using 1 μg peptide/well for coating. Specific antibodies were detected using goat anti-mouse peroxidase-conjugated IgG (KPL, Gaithersburg, MD) at 1 μg/ml, and the reaction was developed with ABTS peroxidase substrate (KPL) and stopped by the addition of 1 × STOP solution (KPL). The absorbance was measured at 405 nm using a Biorad plate reader.

### 2.5 Competitive ELISA

Streptavidin coated 96-well ELISA plates were coated with 1 μg/well biotinylated peptide B as previously described (Major et al., 1999). Equal amounts of mAb (12 ng in 100 μl PBS containing 5% milk) were added to each well with increasing amounts of competitor peptide (from 0 to 0.8 μg). After 1 hr incubation at room temperature and washing, secondary antibody was added. The substrate reaction and absorbance reading were performed as described above.

### 2.6 Affinity Measurements by Octet

Binding kinetics of mAb41 Fab to selected peptides were measured using a Fortebio Octet Red 96 instrument (Menlo Park, CA). All assays were performed in 1 × kinetic buffer (Fortebio). Biotinylated peptides were loaded onto streptavidin biosensors (Fortebio) for 100 s and quenched with biocytin. A two-fold dilution series of antibody Fab (prepared using a mouse IgG1 kit, Pierce) was used as analyte. Association was performed for 300 s and dissociation for 900 s. Binding constants were obtained by fitting sensorgrams with a 1:1 model using ForteBio Data Analysis Software.

### 2.7 Mouse Immunizations

Mice were housed and immunized in the Center for Biologics Evaluation and Research (CBER) Division of Veterinary Services facilities. Studies were carried out in strict accordance with the recommendations in the Guide for the Care and Use of Laboratory Animals of the National Institutes of Health (NIH) and were performed under protocols approved by the CBER Institutional Animal Care and Use Committee. Groups (n = 4–5 animals) of ~ 3-month-old BALB/c mice were immunized subcutaneously with 10 μg peptide emulsified in Complete Freund's Adjuvant as well as intraperitoneally with 10 μg of peptide formulated with Alum. For both routes of administration, a promiscuous polio T-helper peptide (10 μg per mouse) (Leclerc et al., 1991) was combined with the HCV-specific peptide to improve immunogenicity for all immunizations. Boosts at 4 and 8 weeks were performed using the same protocol with the exception of the use of the Incomplete Freund's Adjuvant for subcutaneous injection. The mice were bled 14 days after the second boost, and the immunological responses were measured as described.

### 2.8 Neutralization Assay

Neutralization assays were performed as previously described (Duan et al., 2010) using cell culture HCV (HCVcc) in Huh 7.5 cells. An HCV GT1a/2a chimeric virus was produced by replacing the structural genes of J6/JFH1(a GT2a clone, a gift from Charles Rice at Rockefeller University) with those of the HCV H77 (genotype 1a) strain (Kachko et al., 2011). Virus stocks were diluted in Dulbecco modified Eagle medium (DMEM) supplemented with 10% fetal bovine serum–1% penicillin–streptomycin–2 mM glutamine to yield ~ 50–100 infectious foci per well in the absence of antibodies. Viruses were mixed with diluted polyclonal serum or naïve serum, incubated at 37 °C for 1 h, and then inoculated into Huh 7.5 cells. After 3 days in culture, virus foci were detected either by immunofluorescence or immunoperoxidase staining (Lindenbach et al., 2005) and counted. Neutralization was determined by comparing the infectivity of the viruses incubated with the polyclonal serum to the infectivity of the viruses incubated with media alone. Experimenters who performed the neutralization assay were blinded.

### 2.9 Affinity Depletion of Peptide-specific Antibodies

A total of 20 μg of biotinylated synthetic peptides were incubated with 100 μl of Dynabeads M-280 Streptavidin (Life Technologies) at room temperature for 1 h. After washing with PBS (pH 7.4), the beads were blocked with 5% milk in phosphate-buffered saline (PBS), and incubated with diluted polyclonal serum at room temperature for 1 h. The beads were pelleted with a magnet stand, and the supernatant was collected for further analysis.

### 2.10 Statistical Analysis

Statistical analysis was performed with GraphPad Prism 6 using an unpaired Student *t* test with a two-tailed p value. Error bars represent the standard error of the mean.

## 3 Results

### 3.1 Development of mRNA Display-HTS for Mimotope Identification

**Fig. 1:**
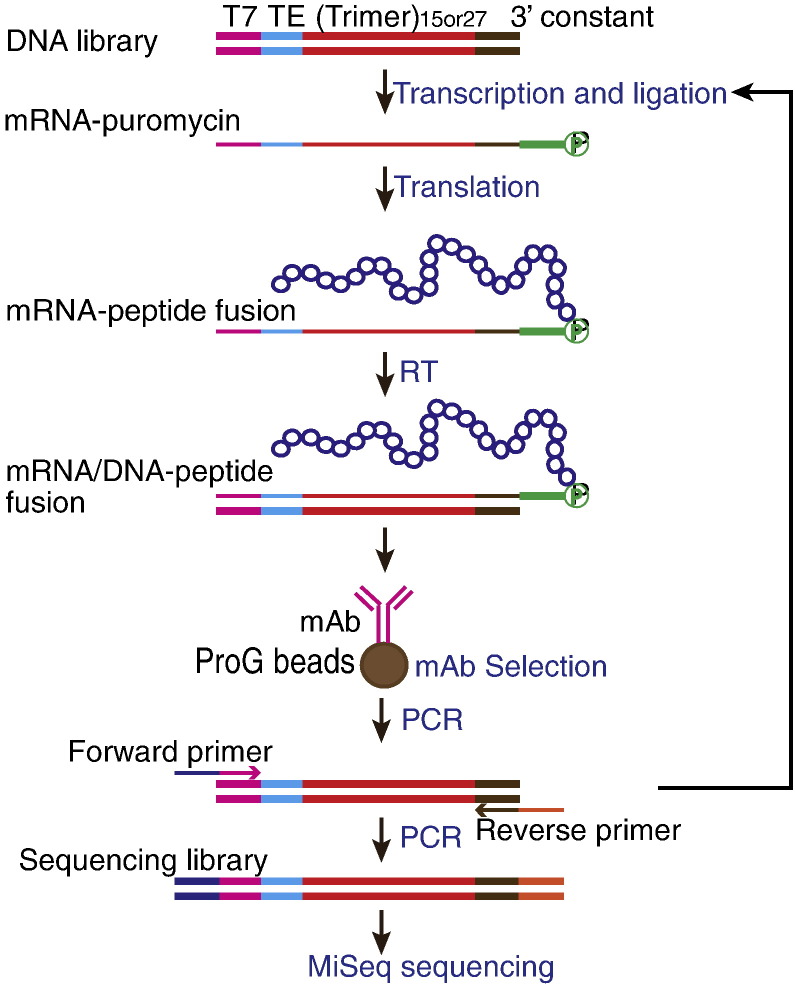
mRNA display selection combined with HTS. The DNA library used contains a T7 promoter (T7), a CMV Translation enhancer (TE), a 15 or 27-mer random region ((Trimer)_15or27_) and a constant region (3′ constant) encoding the peptide QLRNSCA. “Trimer” represents the mixture of 20 trimer (codon) phosphoramidites (Glen Research), each encoding one amino acid. In vitro transcription, ligation to a puromycin linker (green), in vitro translation and reverse transcription (RT) were performed as described in the Materials and methods section. mRNA/DNA–peptide fusions were applied to protein G magnetic beads (ProG beads) complexed with monoclonal antibodies (mAb) for selection. The regenerated DNA library was converted to an Illumina sequencing library by PCR amplification using forward and reverse primers containing Illumina adapters and subjected to MiSeq sequencing.

To validate our experimental system, we first performed mRNA display selection using FLAG M2 mAb as “selection antibody” using a 15-mer library. After each round of selection, nucleic acid sequences linked to the peptides that remained bound to the M2 mAb were PCR-amplified and subjected to Illumina MiSeq HTS sequencing (Fig. 1). We deduced peptide sequences from the HTS results, and ranked the peptides by their frequency within the HTS run as a reflection of their relative affinity for the selection antibody. After 2 rounds of selection with FLAG M2 mAb, the consensus motif DYKXXD homologous to the FLAG epitope (DYKDDDDK) was readily identified among the most frequent peptide sequences obtained (Figure S1), indicating a valid experimental system. Consistent with a recent report (Olson et al., 2012), these results showed that HTS provided a large number of sequences for pattern identification, and reduced the number of mRNA display selection rounds needed to identify peptide binders compared to conventional mRNA display using a low-throughput sequencing method.

### 3.2 mRNA Display-HTS Identifies Motif Patterns That Were Not Detected by Phage Display

**Fig. 2:**
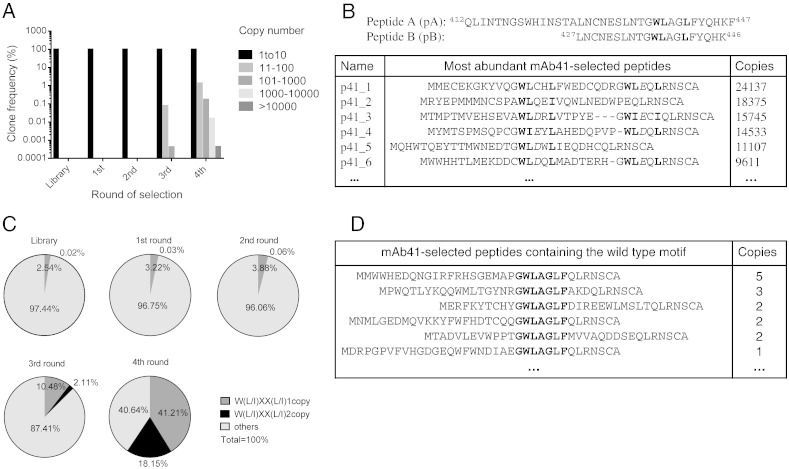
Peptides identified by mRNA display-HTS using HCV mAb41. (A) Clone frequency distribution of the unique peptides with different copy numbers for the input 27-mer library, and 1st to 4th rounds of selection. For each peptide pool obtained after one round of selection, sequences were ranked and divided into different groups based on their copy numbers (1–10, 11–100, 101–1000, 1001–10,000, and > 10,000). The clone frequencies of the unique peptide sequences in each group were calculated and graphed. The total reads were as follows: library (2.3 × 10^6^), 1st round (1.7 × 10^6^), 2nd round (1.5 × 10^6^), 3rd round (1.3 × 10^6^), and 4th round (3.1 × 10^6^). (B) Sequences of the wild type peptide A (pA) and peptide B (pB) from the E2 protein (aa 412–447) of HCV GT1a, and the most abundant peptides selected by mAb41 and their copy numbers are listed. QLRNSCA is the constant region present in all peptides. Dashes are introduced to display sequence alignments. The W(L/I)XX(L/I) motifs are in bold. Acidic residues are italicized. (C) Frequencies of peptides containing 1 or 2 copies of the W(L/I)XX(L/I) motif after each round of selection. (D) Peptides that show a perfect match to the wild type motif (GWLAGLF) were present among peptides selected by mAb41 but with far lower copy number. (B) and (D) are among ~ 3.1 million total sequences obtained after 4th round of selection.

We next performed mRNA display using a 27-mer library against HCV mAb41, for which a previous phage display experiment identified a WL binding motif that aligns with the W^437^ and L^438^ residues in the wild type sequence pA of the HCV GT1a E2 protein (Duan et al., 2012). We carried out four rounds of mRNA display selection and sequenced the selected peptides by HTS after each round. The clone frequency distribution (Fig. 2A) indicated an increase in the abundance of certain unique peptides after the 3rd round of selection, which followed a pre-clearing step with protein G beads in the absence of the selection antibody, indicating enrichment of these peptides. A fourth round of selection further enriched the most common sequences selected in the third round. Identified peptides were ranked by copy number (Fig. 2B), and named based on their rank (with peptide p41_1 being the most abundant peptide binder). The most abundant mRNA display-enriched peptides show a W(L/I)XX(L/I) motif, which aligns with W^437^, L^438^ and L^441^ residues in the wild type sequence. The W(L/I) residues within the motif are similar to the WL identified by previous phage display selection. Different from the phage display results, mRNA display often identified a second (L/I) residue within this motif that aligns with L^441^ (Fig. 2B–C). Although the frequency of W(L/I)XX(L/I) (2.54%, 57,925 reads containing W(L/I)XX(L/I) among 2,278,952 total reads) in the original 27mer library was a bit higher than its expected frequency (1.65%) within a random library, due to an unintentional bias towards inclusion of tryptophan-encoding codons in the commercially prepared input library, a ~ 60% of selected peptides from the 4th round of selection contained at least one copy of W(L/I)XX(L/I) (Fig. 2C), indicating preferential selection of mAb41 for this motif.

Examination of all sequences with copy number exceeding 100 (~ 2000 the most abundant unique peptides) obtained from mRNA display identified additional features including a preference for acidic residues at the position of the first X (Fig. 2B and Figure S2A) and the upstream G and downstream F residues (originally present in the wild type sequence) in some selected peptides (Fig. 2B and Figure S2). No other homology to the wild type E2 sequence was noted outside of the GWLXXLF motif within the selected 27-mers (Fig. 2B). Similar results were obtained when mRNA display selections against mAb41 were carried out using a 15-mer library, for which both W(L/I) and W(L/I)XX(L/I) motifs were identified (Figure S3). The identification of two complete copies of the W(L/I)XX(L/I) motif using the 27mer library most likely is due to its longer length, permitting inclusion of peptides with more than one motif.

mRNA display-HTS of mAb41 also preferentially selected peptides with tandem copies of the W(L/I) or W(L/I)XX(L/I) motifs (Fig. 2B–C and Figure S3), a distinct feature that was not previously detected by phage display. The downstream motif often overlapped the QLRNSCA sequence that corresponds to the constant region of the mRNA display peptides. For peptides selected using the 27-mer library, the percentage of peptides containing tandem copies of W(L/I)XX(L/I) motifs increased over each selection round, especially after the 3rd round of selection, when the percentage of the total number of peptides with the tandem W(L/I)XX(L/I) motif increased thirty five-fold from 0.06% (881 reads with tandem W(L/I)XX(L/I) among 1,455,481 total reads) in the 2nd round to 2.11% (27,300 reads with tandem W(L/I)XX(L/I) among 1,292,412 total reads) in the 3rd round (Fig. 2C). Peptides with a single copy of the wild type sequence (GWLAGLF) were also selected by mAb41, but at much lower copy numbers (Fig. 2D), indicating a lower degree of enrichment.

### 3.3 Selected Peptides Bind to the Selection Antibody mAb41 With High Affinity

**Fig. 3:**
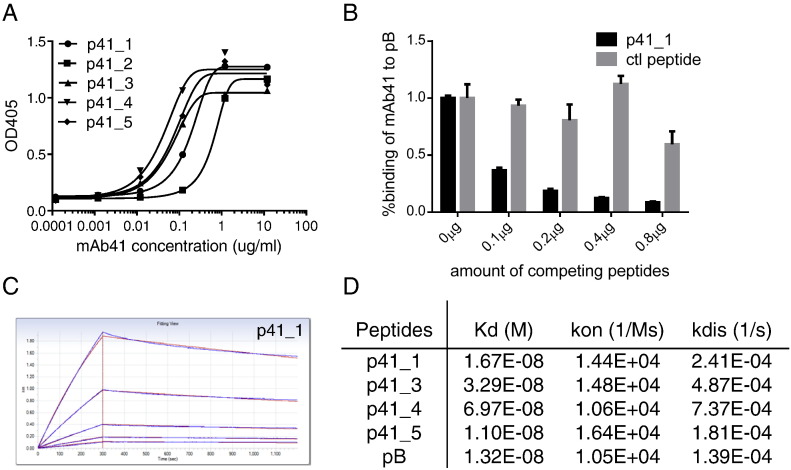
Binding of selected peptides to the selection mAb41. (A) Binding of synthetic peptides to mAb41 at various concentrations. Biotinylated peptides were added to streptavidin-coated microtiter plates. mAb 41 was applied as the primary antibody in a 10-fold dilution series. HRP-conjugated goat anti-mouse antibody was used as the detection antibody. Results shown are the mean of two replicates performed in one of three independent experiments, each of which showed similar results. (B) Competition for binding to mAb41 between p41_1 and wild type pB. 1 μg biotinylated pB was attached to streptavidin-coated plates. 12 ng of mAb41in 100 μl PBS containing 5% milk was added in the presence of increasing amount of p41_1 as indicated. The bound mAb41 was detected using an HRP-conjugated goat anti-mouse antibody. An unrelated peptide from the mRNA display library was used as a negative control. Mean values are graphed and error bars represent SEM of sample replicates. (C) Measurement of binding affinity of selected peptides to mAb41 by Octet RED. Biotinylated peptides were immobilized on Streptavidin biosensors (Fortebio) and Fabs of mAb41 were used as analyte in a 2-fold dilution series ranging from 125 nM to 7.8 nM. Sensorgram data for peptide p41_1 are shown. (D) Binding constants of mAb41-selected peptides to mAb41 Fabs were obtained by fitting sensorgrams (blue) with a 1:1 model (red) using ForteBio Data Analysis Software. p41_2 is not included due to unsatisfactory curve fitting.

The five most abundant selected peptides (p41-1 to p41-5, Fig. 2B) were chemically synthesized for further analysis. By ELISA, mAb41 showed concentration-dependent binding to the mRNA display-selected peptides (Fig. 3A). Peptide p41_1, the most abundant peptide selected by mAb41, was used as a competitor of wild-type peptide B (pB, a shorter version of pA, Fig. 2B) for binding to mAb41 to confirm that the selected peptides interact with the antigen-binding sites of the selection antibody. Increasing amounts of p41_1 inhibited binding of mAb41 to immobilized pB (Fig. 3B). This binding was not appreciably affected by a negative control peptide from a mRNA display library that is not enriched by mAb41 (Fig. 3B), implying that p41_1 binds to the same antigen binding site on mAb41 as does wild type pB. Similar results were obtained using p41_3 as competitor (Figure S4A). Using the Octet binding assay, dissociation constants (K_d_) of mAb41 Fabs with mAb41-selected peptides (with the exception of p41_2 which could not be evaluated due to unsatisfactory curve fitting) were within the 11–70 nanomolar range, similar to the affinity of mAb41 Fab fragments (K_d_ = 13 nM) for wild type pB (Fig. 3C–D and Figure S4B–E).

The acidic residues at the position of the first X in most of the W(L/I)XX(L/I) motifs were evaluated for their contribution to mAb41 binding. By ELISA, an E to A mutation in the second W(L/I)XX(L/I) motif of p41_1(WLEQL) modestly reduced mAb41 binding affinity as compared with peptide p41_1 (Figure S5A,B), whereas substitution of an acidic residue in the corresponding position of p41_2 (a Q to E mutation in WLQEI) conferred an increased binding affinity (Figure S5A, C). Thus, this non-wild-type acidic residue likely plays a role in peptide binding to mAb41.

### 3.4 mRNA Display-selected Peptides Can Elicit mAb41-like Binding and Neutralizing Activity

**Fig. 4:**
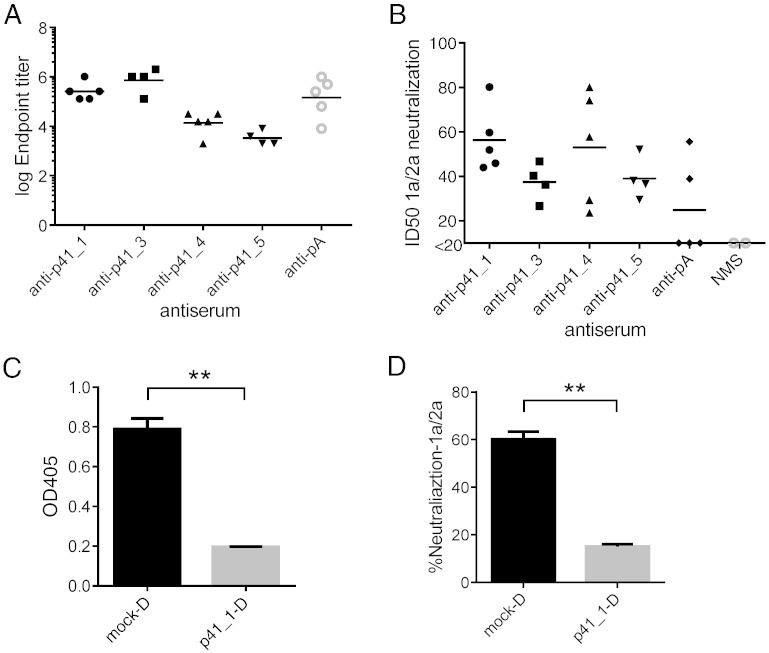
Neutralization of HCVcc GT1a/2a chimeric virus by polyclonal sera from mice immunized with selected peptides. (A) ELISA endpoint titers of polyclonal sera against immunizing peptides. Mice were immunized with peptides as indicated (n = 4 or 5 for each peptide). Polyclonal sera after the second boost were measured for ELISA endpoint titers. (B) Neutralizing ID50 titers of cell culture HCV GT1a/2a chimeric virus by antisera against selected peptides (n = 4 or 5 mice for each peptide). For mice with ID50 < 1:20, 1:10 was used in the mean ID50 calculation. Results shown are the mean of two replicates performed in one of three independent experiments, each of which showed similar results. (C) Anti-p41_1 sera were either mock depleted (mock-D) or depleted using peptide p41_1 (p41_1-D). Depleted sera (1:1000 dilution) were tested for binding to p41_1 by ELISA. (D) HCVcc 1a/2a chimeric virus neutralization after p41_1 depletion of anti-41_1 sera (dilution 1:40). (C) and (D): Similar results observed in n = 3 samples; results from the mouse serum with the strongest neutralizing activity are shown. (Error bars represent SEM of sample duplicates. *p < 0.05, **p < 0.01, Student *t* test, unpaired).

Since the selected peptides bound mAb41 with high affinity, we hypothesized that these peptides might mimic native antigen in presentation of critical epitopes to the immune system and thus might be useful in inducing immune responses that resemble mAb41. We immunized BALB/c mice (n = 4 or 5 for each immunogen) with the top five peptides selected by mAb41. Polyclonal sera were named after the immunizing peptides, i.e. anti-p41_1 is the serum obtained from mice immunized with p41_1. Because mAb41 was cloned from mice previously immunized with pA peptide (Fig. 2B), we immunized mice with the original wild type pA as a control. Polyclonal sera from immunized mice showed binding reactivity to the immunizing peptides with titers in the 1:10^4^ to 10^5^ range for most animals (Fig. 4A), with the exception of p41_2, which showed lower titers (below 2 × 10^3^) and were therefore excluded from further analysis. Since mAb41 specifically neutralizes HCV GT1a, we tested the capacity of these polyclonal sera to neutralize HCV GT1a in vitro using 1a/2a chimeric HCVcc.

We screened antisera from mice immunized with each peptide and found that polyclonal sera against mAb41 selected peptides showed HCV GT1a neutralizing activity in a dose-dependent manner (Figure S6A-D). All of the mice immunized with the mimotope peptides developed neutralizing antibodies against the 1a/2a chimeric HCVcc. The average neutralization titers (ID50) were 1:56 (anti-p41_1), 1:37 (anti-p41_3), 1:53 (anti-p41_4) and 1:43 (anti-p41_5), while naïve mouse serum (NMS) had an undeterminable ID50 (< 1:20) (Fig. 4B). Only 2 out of 5 mice immunized with pA developed detectable neutralizing antibodies against the 1a/2a chimeric HCVcc (Fig. 4B and Figure S6E). To evaluate the specificity of the observed neutralizing activity, anti-p41_1 sera were studied in depletion experiments. The p41_1-specific binding activity was depleted to 25% of original levels (p = 0.0077, as assessed by ELISA) from sera by adsorption to bead-immobilized peptide p41_1 (Fig. 4C). The corresponding neutralizing activity of the depleted sera was also reduced to 25% of original levels (p = 0.002 compared to mock-depleted) when tested in cell culture with 1a/2a chimeric HCVcc (Fig. 4D), indicating that the observed neutralization was specific for the immunizing peptide.

**Fig. 5:**
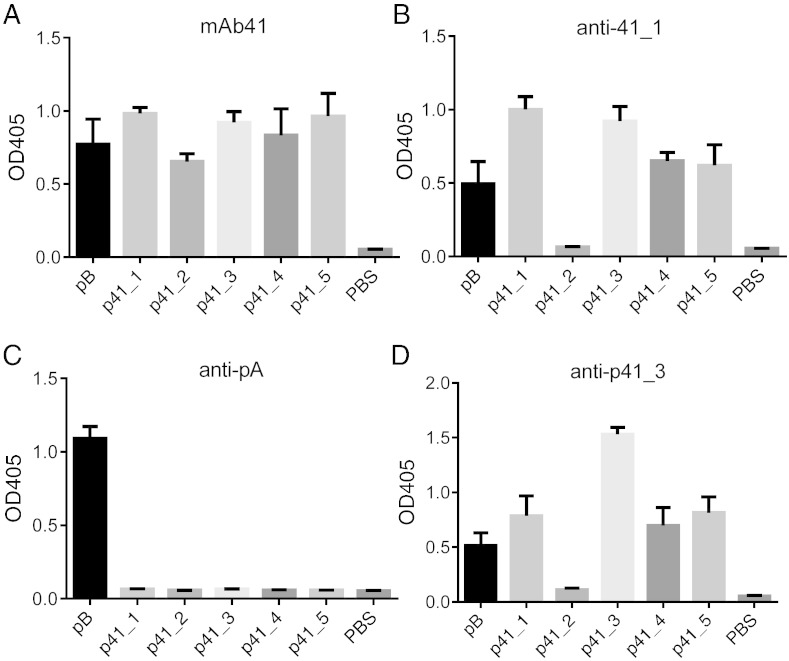
Characterization of the binding reactivity of anti-p41_1 and anti-p41_3 antisera. Biotinylated peptides were immobilized onto streptavidin-coated plates. Binding of (A) mAb41 (1.2 μg/ml), (B) anti-p41_1, (C) anti-pA, and (D) anti-p41_3 (1:1000 dilution, n = 3 for (B–D)) to pB and mAb 41-selected peptides (p41_1 to p41_5) were measured by ELISA. Mean values are graphed and error bars represent SEM of technical replicates for (A) mAb41, and biological replicates for antisera (B–D), with each biological replicate having technical duplicates.

To compare binding characteristics of peptide-elicited antibodies with those of mAb41, we evaluated anti-p41_1 antisera, anti-pA antisera, and mAb41 for their binding reactivity to pB and to the top peptides selected by mAb41. Since peptides p41_1 to p41_5 were enriched by mAb41 through multiple rounds of selection, as a group they likely reflect the structure of the mAb41 antigen binding site and thus were used as signature peptides for detecting mAb41-like antibodies. As expected, mAb41 bound to all the peptides in the ELISA (Fig. 5A). Anti-p41_1 antisera showed a similar binding profile to that of mAb41 when tested against the other mimotope peptides with the exception of p41_2 (Fig. 5B). In contrast, anti-pA sera from the immunized mice showed no detectable binding to any of the top 5 peptides selected by mAb41 and only bound to pB (Fig. 5C). These results suggest that anti-p41_1 sera contains mAb41-like antibodies, while in anti-pA sera, this population is not dominant. Anti-p41_3 antisera showed a binding profile similar to that of anti-p41_1 (Fig. 5D). The reason for the different behavior of p41_2 from that of other mAb41-selected peptides in these assays remains unknown, but may be related to its relatively low binding affinity to mAb41 as assessed by ELISA (Fig. 3A).

### 3.5 mRNA Display-HTS-selected Mimotopes Induce Mainly Motif-specific Antibodies

**Fig. 6:**
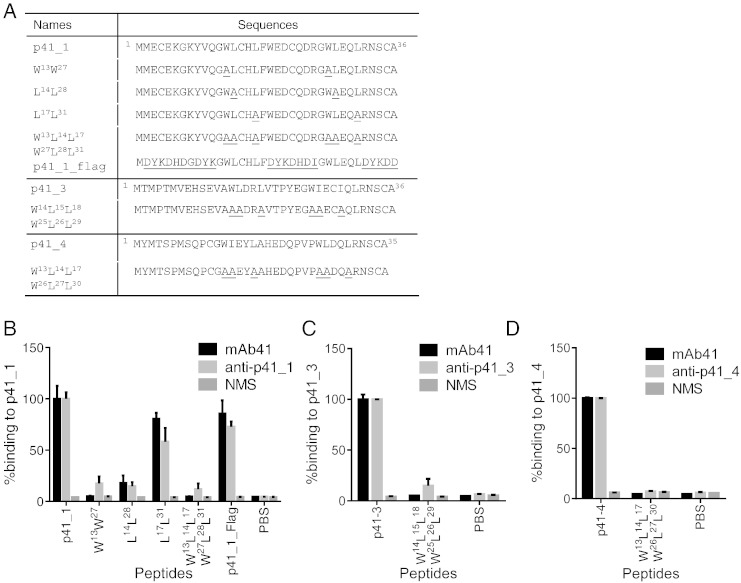
Identification of residues in selected peptides that are important for anti-peptide antiserum recognition. (A) A list of p41_1 mutant peptide sequences is shown. Residues within the tandem repeat WLXXL motif were mutated either individually or in combination to alanine (underlined). A p41_1_flag hybrid peptide retains the segments in p41_1 that contain the duplicated WLXXL motif and the upstream G and downstream F residues, while the remaining residues are replaced by the flag epitope (underlined). p41_3, p41_4 and the corresponding mutant peptides that carry alanine mutations (underlined) in the tandem WLXXL motif are also shown. (B) Binding of mAb41, anti-p41_1 (n = 3) and NMS to p41_1 mutant peptides. (C–D) Binding of mAb41, anti-peptide antisera and NMS to the p41_3 mutant peptide W^14^L^15^L^18^; W^25^L^26^L^29^ (C), and to the p41_4 mutant peptide W^13^L^14^L^17^; W^26^L^27^L^30^ (D). ELISA was performed as described in Fig. 5. Binding reactivities to mutant peptides were graphed as percent binding to the original peptides. Mean values are graphed and error bars represent SEM of technical replicates for mAb41 and biological replicates for antisera, with each antiserum having technical duplicates.

To further characterize the binding properties of polyclonal antibodies induced by mAb41-selected peptides, we assessed binding of polyclonal anti-p41_1 sera to a series of p41_1 mutant peptides (Fig. 6A). We mutated the key residues in the duplicated WLXXL motif to alanine either individually or in combination, and tested binding of anti-p41_1 antisera and mAb41 to these mutant peptides. To evaluate the contribution of residues outside of the motif to the antibody response against p41_1, we also designed a hybrid peptide (designated p41_1_flag) in which a GWLXXLF motif was preserved and other residues were replaced by the FLAG^TM^ epitope sequence (Fig. 6A). The overall binding profiles to p41_1 mutant peptides were similar between anti-p41_1 and mAb41 (Fig. 6B), further indicating that anti-p41_1 contains mAb41-like antibodies. Both antibodies show significantly reduced binding to mutant peptides containing either the W^13^W^27^ mutations or the L^14^L^28^ mutations. The L^17^ L^31^ mutations also resulted in a modest reduction in binding to both antibodies. Mutations of all the motif residues (as indicated by peptide W^13^ L^14^ L^17^W^27^ L^28^ L^31^) resulted in almost complete abrogation of binding to both antibodies to a level similar to the signal obtained with NMS. p41_1 thus preferentially induced antibodies to the specific WLXXL binding motifs and did not induce large amounts of nonspecific antibodies to other residues or motifs present within the peptide. The binding of mAb41 and anti-p41_1 to the p41_1_flag hybrid peptide remained strong (Fig. 6B), indicating that the residues surrounding the GWLXXLF motifs are likely not contact residues in antibody binding, and further indicating that the p41_1-induced antibodies are mainly directed against the specific motifs. Anti-p41_1 and mAb41 show a trend towards reduced binding to the hybrid peptide relative to p41_1, suggesting that the surrounding non-motif residues contribute to an optimized presentation of the core motifs. Reduced binding of antisera to the corresponding mutant peptides was also observed for p41_3 and p41_4 when the WLXXL motifs were mutated (Fig. 6A, C–D), indicating that the ability to induce a mAb41 epitope-focused immune response is a common feature shared by the top peptides selected by mAb41.

## 4 Discussion

The use of a high complexity selection library with massive data output allowed mRNA display-HTS to unlock the intrinsic information embedded in mAb antigen binding sites. Selected peptides identify unique motif patterns, and as a whole, represent the binding specificity of mAb41. The faithful capture of information intrinsic to the selection mAb by the selected peptides was further manifested by the ability of these peptides to induce antibodies that mimic both the epitope-specific binding and the neutralizing activity of the original mAb. The present findings support the general use of mRNA display-HTS as a powerful approach in antigen identification for reverse-engineering of vaccines.

The high library complexity, afforded by mRNA display-HTS allowed identification of distinct features not identified in a previous phage display experiment, including the preference for a tandem copy of the W(L/I)XX(L/I) motif and acidic residues at certain positions. The importance of key residues for antibody interaction was confirmed through mutational analyses. The selection of peptides with a tandem copy of the motif may be due to combined effects of high affinity and avidity. The increased avidity implied by selection of a duplicated pattern might be related to interaction of two epitope motifs with two adjacent antigen binding sites of the selection antibody immobilized on the protein G beads. The peptides selected within the mRNA display experiment showed in vitro affinity for the monovalent mAb41 Fabs similar to that of the wild-type sequence in the Octet binding assay, even though the only common sequence among the peptides was the W(L/I)XX(L/I) motif. This binding was strong for the peptides containing single (p41_5) or tandem W(L/I)XX(L/I) repeats (p41_1, p41-3 and p41_4). The Octet system, which used monovalent mAb41 Fabs, may not have been capable of detecting a significant increase in affinity for peptides with a second motif. These data confirm that the presence of this motif alone is sufficient for high affinity binding to the mAb41 and reinforces the application of the mRNA display technique for selection and analysis of antibody epitopes.

We studied the most abundant peptides in the mRNA display-HTS library for their ability to induce mAb41-like properties. p41_1 and p41_3 induced polyclonal sera with mAb41-like binding activity, preferentially to the specific WLXXL binding motifs, more effectively than did the original wild type pA. All antigen-antibody binding, including that of antibody recognizing linear epitopes, involves a particular conformation of the antigen. Since these peptides underwent several rounds of affinity-selection by mAb41, they (and other peptides selected by mRNA display) likely possess complementarities optimized for the mAb41 binding site, and thus may have a propensity to adopt a conformation more closely resembling that of the native antigen in the E2 protein than does pA. Differences in solubility precluded comparison of peptide secondary structure by circular dichroism; however, bioinformatic analysis using TASSER predicted alpha-helical structures in the regions containing the W(L/I)XX(L/I) motif both of the mRNA display-selected peptides and of pA (data not shown), It is unlikely that disulfide bonds could have contributed to peptide secondary structure during the mRNA display selection because the preceding reverse transcription step was performed in the presence of DTT.

We did not observe clear correlations between relative mAb41 binding affinities of peptides as assessed by different methods (the mRNA display selection, or in Octet or ELISA assays) and virus neutralization of induced polyclonal serum, suggesting that there may be differences among the binding interactions detected by these methods. However, all methods indicated strong mAb41 binding of p41_1, p41_3, p41_4, and p41_5. p41_2 showed both relatively lower mAb41 affinity by ELISA and weaker neutralizing immune responses. Improved neutralizing responses induced by mAb41-selected peptides than pA could also be mediated by the duplicated W(L/I)XX(L/I) motif and/or the acidic residues that allow a more favorable presentation of the epitope. It is possible that pA presents the critical residues in a less favorable manner either due to the loss of optimal conformation when removed from its native context (Fibriansah et al., 2014), or the lack of mAb41 preferred features (duplicated W(L/I)XX(L/I) motif and acidic residues). This is evidenced by the absence of detectable mAb41-like antibodies in anti-pA polyclonal sera, despite the fact that this peptide was used for the initial immunizations that resulted in the isolation of mAb41 (Duan et al., 2012). These results suggest that mAb41 was a rare species in the antibody pool originally induced by pA. Although some anti-pA antisera showed GT1a neutralizing activity, this activity could have been mediated by non-mAb41-like antibodies, because pA also contains other neutralizing epitopes (Duan et al., 2012). In contrast, p41_1 and p41_3 induced a higher frequency of mAb41-like antibodies, targeting specific residues within the critical motif, suggesting a more favorable presentation of the WLXXL epitope than in pA. These results indicate that mimotopes identified by mRNA display-HTS have potential as improved vaccine antigens that shift the immune response towards the production of epitope-specific antibodies with desired activity, achieving a result similar to that of other epitope-focused vaccine design approaches (Correia et al., 2014). The mRNA display-HTS approach could also be applied to identification of linear mimetics for mAbs against conformational epitopes. Due to the structural complexity of conformational epitopes, linear peptides may not be able to recapitulate the full three dimensional structure of the original antigens. Using more complex libraries (for example, of 80mer (Keefe and Szostak, 2001, Cho et al., 2000)) could help to address this limitation.

We chose to study mAb41 because it is a potent neutralizing antibody that arises from an antigenic region that can induce both neutralizing and non-neutralizing antibodies, providing a basis for investigating whether mAb41 mimotopes could induce immune responses with mAb41-like properties, including neutralization. MAb41-based mimotopes by themselves may have limitations as HCV vaccines, due to the genotype-specificity of mAb41 neutralization. Although peptides are generally not considered ideal vaccine antigens, due to their ability to adopt many conformations, we propose that such antigens have potential utility as priming antigens to induce initial immune responses with desired attributes, setting the stage for boosting with wild type proteins or viral particles. mRNA display-selected peptides, carrying information specific to neutralizing antibodies and the ability to direct epitope-targeted immune responses, would potentially be more useful as priming antigens than would wild type peptides. The magnitude of the immune response observed after immunization of mice with mAb41-selected peptides, is similar to that of some effective human vaccines, even with a single epitope. While antibody titers needed to protect against HCV infections are not known, neutralizing titers of 1:10 (or lower) are generally considered correlates of efficacy for rubella and measles vaccines (Hilleman et al., 1967). The magnitude of the neutralizing titers induced after selected peptide immunization could potentially be further improved using other antigen presentation techniques (e.g., by addition of scaffold proteins or presentation of antigens on the surface of virus-like particles) or adjuvants, or by using a mixture of mRNA-display-selected mimotopes to present multiple epitopes to the immune system.

The present study shows that previously inaccessible information encoded in mAbs can be unlocked using mRNA display, and demonstrates the promise of mRNA display systems in antigenic characterization in support of reverse-engineering of vaccines. In their totality, peptides selected from a random library by mRNA display characterize a mAb's binding specificity and provide information about antigens that can induce polyclonal humoral immune responses that mimic the original mAb. This improved understanding of mAbs could lead to better vaccine antigens, improved characterization of monoclonal antibody-based products, and insights into the mechanism of action of monoclonal antibodies (mAbs).

## Declaration of Interests

The authors have no conflict of interests.

## Author Contributions

NG, HD, MM, and PK designed the study. NG, HD, AK, BK, MM and PK performed data collection and analysis. NG, HD, AK, MM and PK interpreted the data. NG, MM and PK wrote the manuscript.

## Funding

This study was funded with intramural FDA funds. As FDA employees, the authors wrote the manuscript and decided to submit it for publication. The authors were not paid to write the article by a pharmaceutical company.
