# Supplementary material for: Reverse Engineering of Vaccine Antigens Using High Throughput Sequencing-enhanced mRNA Display
Source: eBioMedicine. 2015 Jun 30;2(8):859–67. doi: 10.1016/j.ebiom.2015.06.021 (PMC4563141; doi:10.1016/j.ebiom.2015.06.021)
Supplement: Supplementary file 1 — Supplementary figures. [file mmc1.pdf]

Supplemental figure legends

Figure S1. Peptides selected by mRNA display-HTS using FlAG M2 mAb. The most abundant peptides selected by FLAG M2 mAb and their copy numbers are shown (among ~1 million total sequences obtained after 2 rounds of mRNA display selection). Residues that are identical to the FLAG epitope DYKDDDDK are in bold.

Figure S2. Acid residues are present at a high frequency adjacent to the W(L/I) motif. Peptide sequences obtained from the 4th round of selection (copy numbers > 100, ~2000 clones) were used for analysis. Sequences that contain the W(L/I) motif were selected and the amino acid residue frequencies were calculated in the flanking regions. Residue W was set as position 0 and its frequency 100%. Background residue frequencies are less than 10% (not shown). Acidic residues D and E show high frequencies at the +2 position. Residue G shows high frequency at position -1, and the F residue also showed a frequency higher than the background at position +5. Both G and F are present in the original wild type sequence

Figure S3. The most abundant peptides selected by mAb41 using a 15-mer library. Copy numbers are among ~1 million total sequences obtained after 2 rounds of mRNA display selection). QLRNSCA is the constant region. Dashes are artificially introduced to show sequence alignments. The W(L/I) or W(L/I)XX(L/I) motifs are in bold. Acidic residues next to the W(L/I) motif are Italicized. The upstream G residue (Italicized) was also identified.

Figure S4. **Binding of selected peptides to mAb41. (A)** Competition for binding to mAb41 between p41_3 and wild type pB. Binding was carried out as described in **Figure. 3**. (**B-E**) Sensorgrams (blue) obtained by Octet RED and curve fitting (red, 1:1 model) for binding of the mAb41 Fabs to peptides (**B**) pB, (**C**) p41_3, (**D**) p41_4, and (**E**) p41_5.

Figure S5. Assessment of the role of the acidic residues in binding of selected peptides to mAb41. (**A**) Biotinylated mutant peptides were synthesized. p41_1_EtoA carries an E to A mutation within the second WLXXL motif of p41_1. p41_2_QtoD carries a Q to D mutation in the WLQEI motif of p41_2. (**B-C**) Binding of the p41_1 (**B**) and p41_2 (**C**) mutant peptides to mAb41 was measured by ELISA in comparison with the original peptides. ELISA was performed as described in **Figure 3** except that mAb 41 was applied as primary antibody in a 2-fold dilution series.

Figure S6. Neutralization of HCVcc GT1a/2a chimeric virus. Polyclonal sera from mice immunized with peptides (**A**) p41_1, (**B**) p41_3, (**C**) p41_4, (**D**) p41_5, (**E**) pA, or (**F**) NMS were tested for HCVcc GT1a/2a neutralizing activities. For each serum sample, four dilutions of 1:20, 1:40, 1:80 and 1:160 were used.

SFig1

Peptides selected by anti-Flag M2	Copies
MQICN**DYKDDD**V**K**NSVQLRNSCA	16
MYKLMDRS**DYK**YA**D**THQLRNSCA	13
MCYGD**DYK**NG**D**LTLQRQLRNSCA	9
MSYPCDIPTF**DYK**MT**D**QLRNSCA	9
MRTRMKNP**DYK**NE**D**CLQLRNSCA	8
**...**	**...**

SFig2

Positions / Residues	X<br>-1	W<br>0	[LI]<br>+1	X<br>+2	X<br>+3	X<br>+4	X<br>+5
W		100%					
L			77.4%			60.6%	
I			22.6%			10.6%	
D				33.7%			
E				28.5%			
G	43.2%						
F							12.2%

SFig3

Peptides selected by mAb41 using a 15-mer library	Copies
M**WI**HG-------*G***WL***E*T**L**TTCSGQLRNSCA	10
MLWYHKCANW---*G***WL**NS**L**QLRNSCA	8
MCDRKSGLLWNY---*G***WL***E*Q**L**RNSCA	6
MKES**WI***D*G**I**WRE---*G***WL***E*Q**L**RNSCA	6
MIWEYNT*G***WL***D*S**I**WVSQLRNSCA	5
M**WL**CWNYDKNKT*G***WL***D*Q**L**RNSCA	5
...	...

SFig4

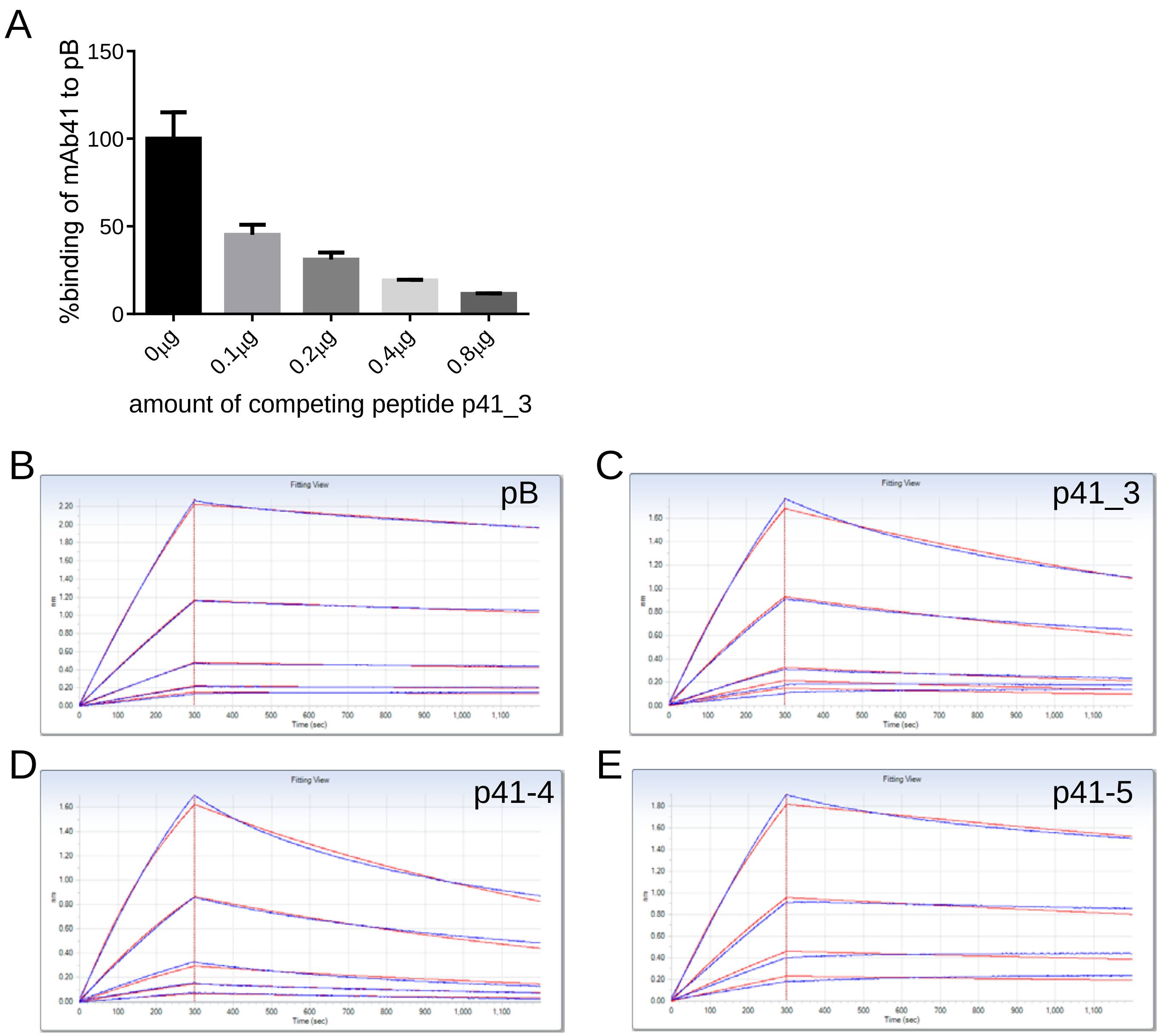

A
%binding of mAb41 to pB
150
100
50
0
0µg
0.1µg
0.2µg
0.4µg
0.8µg
amount of competing peptide p41_3
B
pB
C
p41_3
D
p41-4
E
p41-5
Fitting View
Time (sec)
nm

SFig5

A

Names	Sequences
p41_1	MMECEKGKYVQGWLCHLFWEDCQDRGWLEQLRNSCA
P41_1_EtoA	MMECEKGKYVQGWLCHLFWEDCQDRGWLAQLRNSCA
P41_2	MRYEPMMMNCSPAWLQEIVQWLNEDWPEQLRNSCA
P41_2_QtoD	MRYEPMMMNCSPAWLDEIVQWLNEDWPEQLRNSCA

B

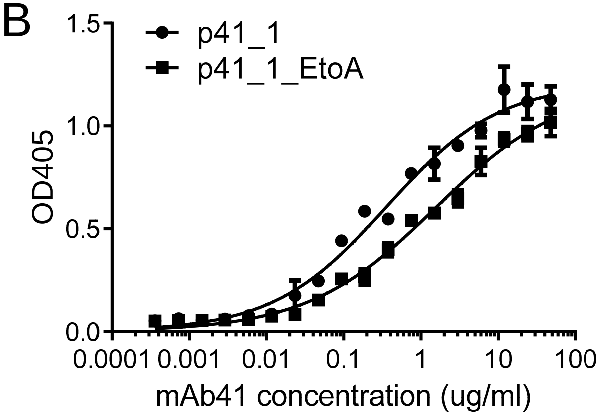

C

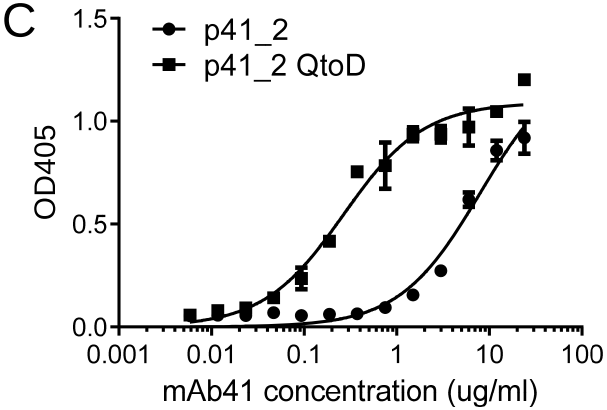

SFig6

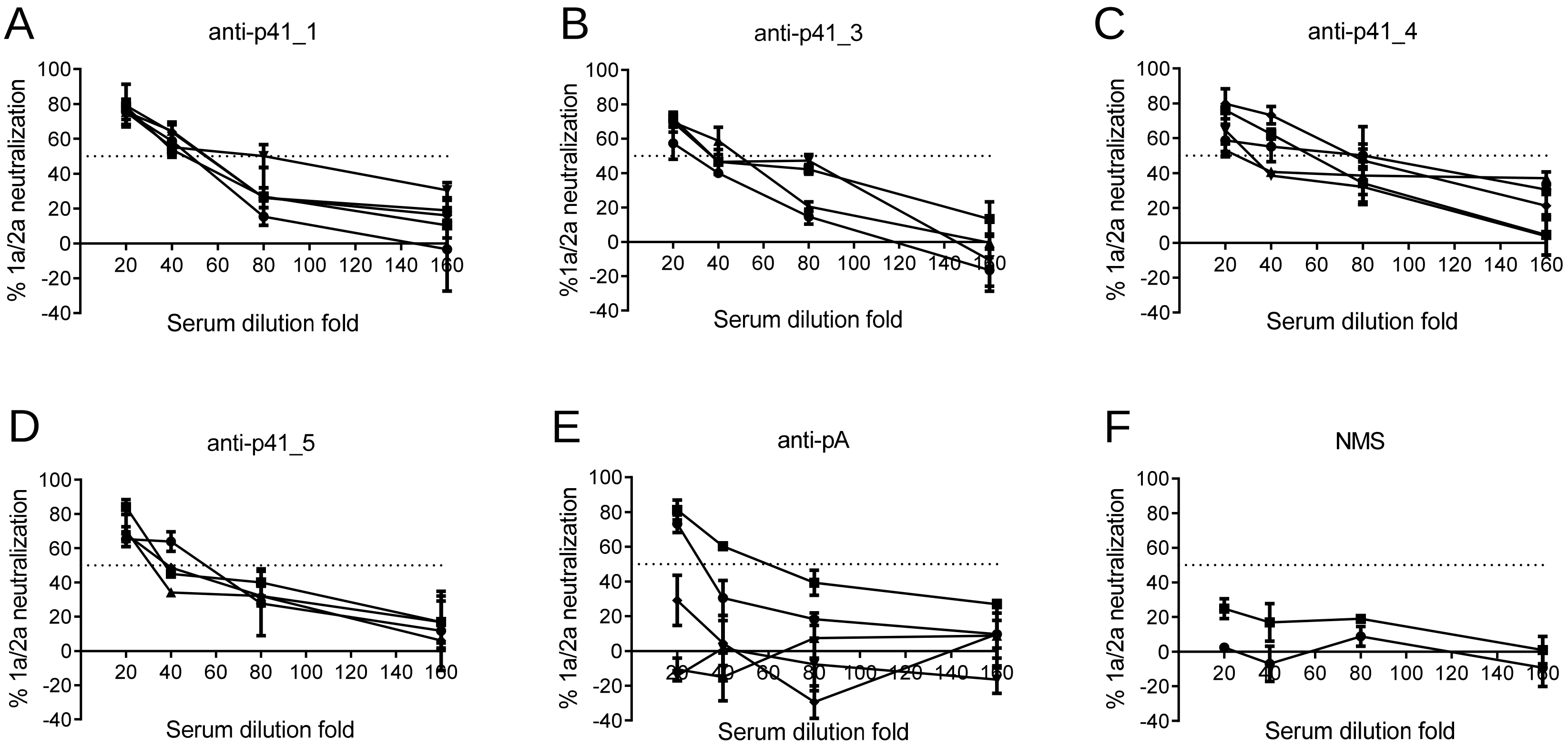

A
anti-p41_1
B
anti-p41_3
C
anti-p41_4
D
anti-p41_5
E
anti-pA
F
NMS
% 1a/2a neutralization
Serum dilution fold
100
80
60
40
20
0
-20
-40
20
40
60
80
100
120
140
160
